# Cytokine Therapy Combined with Nanomaterials Participates in Cancer Immunotherapy

**DOI:** 10.3390/pharmaceutics14122606

**Published:** 2022-11-26

**Authors:** Heping Lian, Shuang Ma, Duoyi Zhao, Wei Zhao, Yan Cui, Yingqi Hua, Zhiyu Zhang

**Affiliations:** 1 Department of Orthopedics, The Fourth Affiliated Hospital of China Medical University, Shenyang 110032, China; 2 Nursing Department, The Fourth Affiliated Hospital of China Medical University, Shenyang 110032, China; 3 Department of Orthopedics, Shanghai General Hospital, Shanghai Jiao Tong University School of Medicine, Shanghai Bone Tumor Institution, Shanghai 200080, China

**Keywords:** immunotherapy, drug delivery systems, cytokine therapy, nanomaterial, combination therapy of cancer

## Abstract

Immunotherapy has gradually become an emerging treatment modality for tumors after surgery, radiotherapy, and chemotherapy. Cytokine therapy is a promising treatment for cancer immunotherapy. Currently, there are many preclinical theoretical bases to support this treatment strategy and a variety of cytokines in clinical trials. When cytokines were applied to tumor immunotherapy, it was found that the efficacy was not satisfactory. As research on tumor immunity has deepened, the role of cytokines in the tumor microenvironment has been further explored. Meanwhile, the study of nanomaterials in drug delivery has been fully developed in the past 20 years. Researchers have begun to think about the possibility of combining cytokine therapy with nanomaterials. Herein, we briefly review various nano-delivery systems that can directly deliver cytokines or regulate the expression of cytokines in tumor cells for cancer immunotherapy. We further discussed the feasibility of the combination of various therapies. We looked forward to the main challenges, opportunities, and prospects of tumor immunotherapy with multiple cytokines and a nano-delivery system.

## 1. Introduction

So far, cancer is still the most severe disease. The treatment methods usually include surgery, chemotherapy, and radiotherapy. Tumor immunotherapy, which inhibits tumor development by activating the immune system, has been considered the fourth most popular tumor therapy [1,2]. The immune escape strategy of tumor cells is regarded as a significant obstacle to immunotherapy for all cancers and provides favorable conditions for tumor progression and immune tolerance. In cancer immunotherapy, drugs activate the immune system against tumor progression and metastasis through enhanced immune responses [3,4].

The earliest records of immunotherapy for cancer can be traced back to ancient Egypt, when some tumors subsided naturally after inflammation [5]. The first to study cancer treatment through the immune system were two German doctors, Fehleisen and Busch, who found that the tumor disappeared after the patient was infected with erysipelas [6,7]. The subsequent considerable development comes from William Coley, who first attempted to use the immune system to treat tumors in 1891 [8,9]. He found some cases of natural remission in cancer patients after an erysipelas infection. He studied in depth the records left by his predecessors and found as many as 47 cases of cancer patients who could not be cured in theory and reported natural remission after acute bacterial infection [5,10]. However, because the proposed “Coley’s toxins” did not have a precise mechanism of action at that time and because of the risk of using highly pathogenic bacteria to infect cancer patients, the research results of Coley were shelved by academic circles until 1967, when Jacques Miller discovered the existence of T cells, he described their functions in *Nature*. People began to pay attention to the immune system [11].

Meanwhile, people also began to figure out how to use immunotherapy to treat cancer. IFN-α was approved for cancer immunotherapy in 1986 [12]. High-dose recombinant IL-2 was approved for metastatic renal cell carcinoma treatment in 1992 and then approved for metastatic melanoma in 1998.

In recent years, some cytokines have been used in various animal cancer models for research [13]. Cytokines are soluble proteins that respond to immune cells by transmitting inflammatory or anti-inflammatory signals, with dual and conflicting signals [14]. Once the cytokine meets the membrane receptor on the target cell, the intracellular signal pathway will be triggered, thus inducing different cells’ survival, activation, and differentiation in the tumor microenvironment (TME). Various cytokines play their roles in the location of the tumor.

Most of the cytokines used in tumor therapy are “pro-inflammatory” factors that enhance the immune system response by stimulating immune cells to modulate the immune microenvironment of tumors. The immune system relies on APC cells to present antigens to immunological effector cells, which act as antitumor agents by secreting antibodies or by direct killing. Due to the immune escape mechanism, adding cytokines to therapy can enhance this antitumor pathway. For example, IL-2 can promote T cell responses, NK and CD4+ cell proliferation, and antibody production by B cells [15,16]; IFN-γ primarily regulates CD8+ and CD4+ T cell immune responses [17]. These properties allow the delivery of cytokines into the tumor microenvironment using drug delivery systems to enhance tumor immunotherapy.

On the one hand, some cytokines, such as IL-4 and IL-8, accelerate the progression of tumors and inhibit immunity. On the other hand, other cytokines have also played a vital role in enhancing the antitumor immune response. Cytokines used in cancer immunotherapy can be divided into the following categories: ① IL-2 Family: IL-2,7,15,21; ② IFN-α; ③ IFN-γ; ④ IL-12; ⑤ TNF; ⑥ colony-stimulating factor (CSF) Family: GM-CSF, Granulocyte (G)-CSF, erythropoietin (EPO), IL-3; ⑦ IL-1 Family: IL-1,18 [18].

In recent years, the involvement of various nanomaterials in tumor immunomodulation therapy has been shown to effectively target tumor tissues, which helps reduce the dose of administered drugs and mitigate adverse effects [19,20]. The application of nanomaterials can avoid degradation of the drug before reaching the tumor and achieve enrichment at the tumor site through enhanced permeability and retention (EPR) effects or active targeting [21]. 

**Scheme 1:**
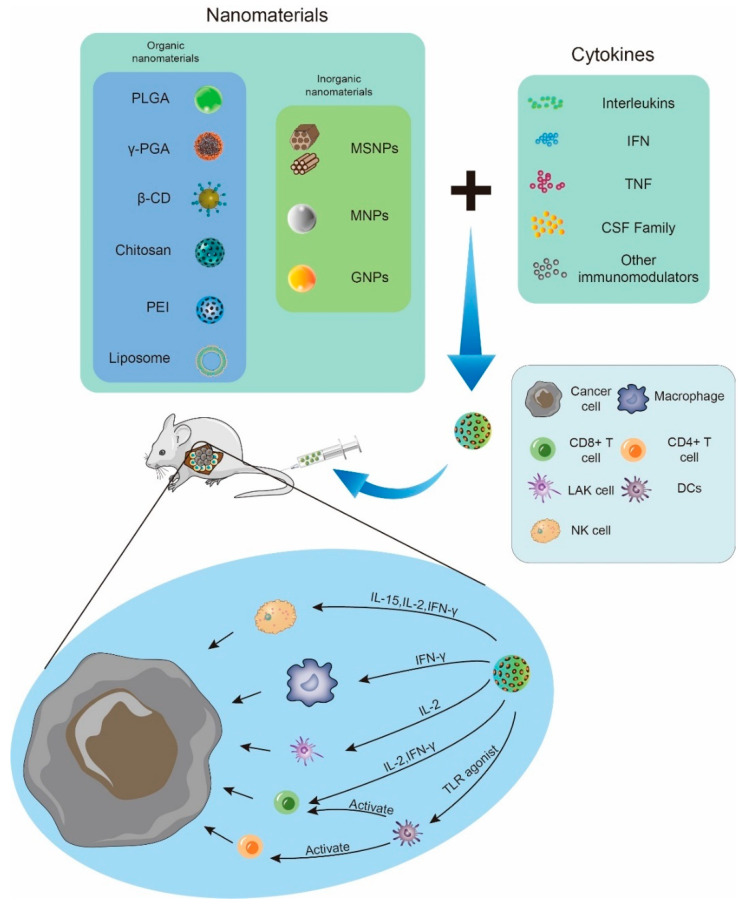
Schematic diagram of nanomaterials loaded with cytokines participating in cancer immunotherapy.

In this paper, we briefly review various nano-delivery systems that can directly deliver cytokines or regulate their expression in tumor cells for cancer immunotherapy (Scheme 1). We further discussed the feasibility of the combination of various therapies. We looked forward to the main challenges, opportunities, and prospects of tumor immunotherapy with multiple cytokines and a nano-delivery system.

## 2. Organic Nanomaterials

After years of exploration, researchers have discovered a variety of organic nanomaterials that can be used to deliver cytokines to target cells. Using these materials to transport cytokines is more efficient than using free drugs. At the same time, because organic materials are easier to modify and process, researchers can change the materials according to different needs to make the materials have other functions. These efforts make cytokines more and more important in cancer immunotherapy. This paper summarizes the existing organic nanomaterials into the following six categories: poly (lactic-co-glycolic acid)-based nanomaterials, poly-γ-glutamic acid-based nanomaterials, β-cyclodextrin-based nanomaterials, chitosan-based nanomaterials, polyethyleneimine-based nanomaterials, and liposome-based nanomaterials (Table 1).

### 2.1. Poly (Lactic-Co-Glycolic Acid)-Based Nanomaterials

Poly(lactic-co-glycolic acid) (PLGA) is obtained from the random ring-opening copolymerization of a cyclic dimer of glycolic acid and lactic acid (1,4-dioxohexa-2,5-dione) [46,47,48]. Different ratios of PLGA can be obtained by adding different proportions of raw materials. For instance, PLGA 50:50 is obtained by synthesizing 50% lactic acid and 50% glycolic acid [49,50]. PLGA is virtually nontoxic to cells, which has led to its early application in biomedicine, and drugs loaded using PLGA have excellent sustained release [51,52,53].

Inspired by the properties of PLGA, Maravajjala, K.S. et al. prepared a pH-sensitive multi-arm PLGA polymer nano-drug delivery system with Paclitaxel (PTX) and Resiquimod (RSQ). They validated the antitumor activity in a mouse breast cancer model [54]. Toll-like receptor (TLR) agonists significantly affect the activation of macrophages. Of all the TLR agonists, RSQ has shown considerable activation of tumor-associated macrophages (TAM) in many studies [55,56,57]. In this study, the reported nanoparticles effectively enhanced the immune response in the tumor microenvironment [54]. At the same time, this multi-arm PLGA polymer showed excellent pH sensitivity, which significantly improved the activity of combined chemotherapy and immunotherapy. In another study, Da Silva, C. and his colleagues used RSQ, CCL20, and PLGA to prepare PLGA nanoparticles by solvent volatilization-extraction, which co-delivered immunomodulators to regulate the inhibitory tumor microenvironment and promote the systemic immune response [23]. They explored the therapeutic effects in a mouse lymphoma model. About 75% of the mice survived for a longer time. Tang, X.D. et al. used PLGA nanoparticles in combination with TLR3/7 ligands to enhance immunogenicity. These ligands are targeted to DCs by DEC-205 antibodies [58]. The experimental results show that the PLGA nanoparticles can target and internalize DC more effectively, stimulate T cells to produce higher levels of IL-12 and interferon-γ in vitro, and upregulate the number of T cells. In a recent study, Mihalik, N.E. et al. achieved tumor targeting and controlled release of GM-CSF by preparing PLGA/PLGA-PEG nanoparticles loaded with GM-CSF [24].

### 2.2. Poly-γ-Glutamic Acid-Based Nanomaterials

Poly-γ-glutamic acid (γ-PGA) is a kind of natural macromolecule compound composed of glutamic acid. Polymer α-PGA is produced by chemical synthesis, while γ-PGA is produced by several bacillus microorganisms [59,60,61]. In synthesizing γ-PGA, alkaline hydrolysis can change its relative molecular weight. In addition, enzymes or ultrasonication can be employed to control the relative molecular weight of γ-PGA [61,62,63]. γ-PGA also has good biocompatibility, biodegradability, and water solubility. There are no adverse reactions in people and animals. The application of γ-PGA in various areas, such as food and medicine, has been extensively studied [60,64]. 

**Figure 1:**
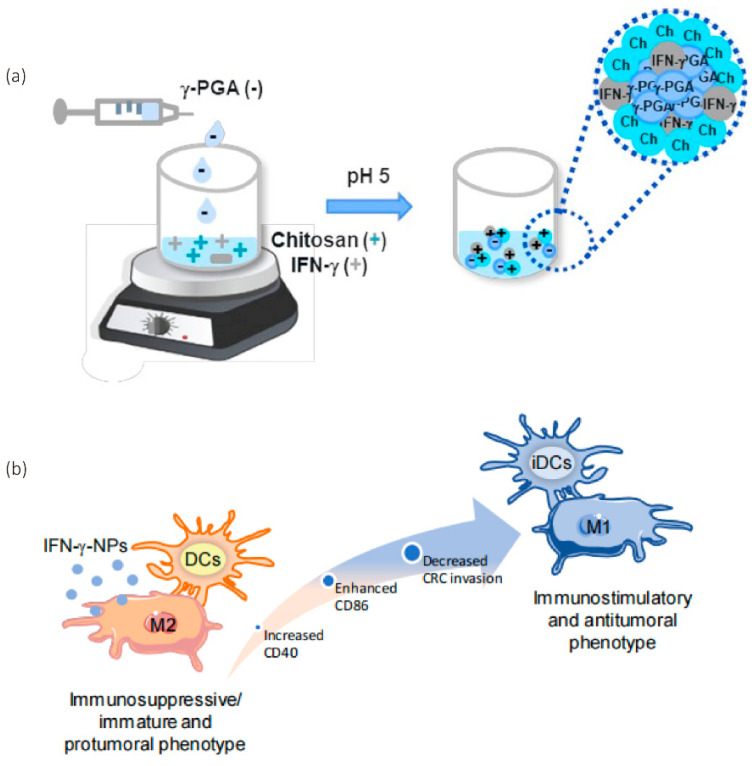
(**a**) Schematic representation of IFN-γ-NPs production and (**b**) the potential synergistic effect of chitosan/y-PGA nanoparticles with immunomodulatory cytokines, such as IFN-γ, for anti-cancer therapy. Reproduced with permission from Castro, F. et al., *Biomaterials Science*; published by the Royal Society of Chemistry, 2019 [25].

Poly-γ-glutamic acid has a regulatory effect on the immune response to CD40 and can effectively enhance APC antigen presentation [65]. In oncology, many studies use γ-PGA nanoparticles loaded with drugs to treat tumors [66,67]. Chitosan/γ-PGA nanoparticle is a widely studied nano-delivery platform (Figure 1). Castro, F. et al. first explored the immune activity of chitosan/γ-PGA nanoparticles [67]. These nanoparticles can enhance DC differentiation, the expression of co-stimulatory molecules, and the production of pro-inflammatory cytokines. After that, they synthesized chitosan/γ-PGA nanoparticles loaded with IFN-γ and tested their immunomodulatory ability [25]. The nanoparticles have the effect of enhancing cytokine secretion by macrophages. In the presence of nanoparticles, IL-6, IL-12, and TNF-α levels significantly increased, while the invasion and migration abilities of colorectal cancer cells were inhibited.

### 2.3. β-Cyclodextrin-Based Nanomaterials

Cyclodextrin (CD) is a kind of natural cyclic oligosaccharide widely used in pharmaceutical and industrial fields. The hydrophilic modification of insoluble drugs can be carried out by cyclodextrin coating; as a result, various CD-based drug loads can be achieved [68]. The low toxicity of cyclodextrin makes it highly biosafe as the raw material for nano-carriers [69,70]. In addition, HP-β-CD was approved for clinical treatment. Many research groups have developed strategies for delivering immunomodulators using β-CD-based nanoparticles.

For the past few years, the β-CD nano-delivery system has been reported to act as an immune adjuvant. This claim was confirmed in a clinical trial in which a panel using β-cyclodextrin as an adjuvant stimulated more CD4+ T cells to produce TNF-α when antigen-stimulated PBMC [71]. In the study of Jiang, J. et al., they synthesized a β-CD-polyethyleneimine nano-delivery system loaded with SB-50524 and adenovirus [34]. The system successfully reduced TGF-β levels in mouse melanoma tissues. It delivered adenoviral vectors carrying the IL-12 gene to tumor sites, achieving high expression of IL-12 at tumor sites, successfully inhibiting tumor growth and prolonging animal survival. Shang, L. et al. designed a nanometer gel loaded with PTX and IL-2 [72]. The system is pH-responsive and can target tumor cells and release IL-2 and PTX simultaneously to achieve chemotherapy combined with immunotherapy for triple-negative breast cancer. Rodell, C.B. et al. designed a covalently cross-linked CD nanoparticle-containing R848. In the colon cancer model of C57BL/6 mice, the nanoparticles successfully targeted TLR agonists to TAM and effectively increased the content of IL-12 in TME [30]. 

### 2.4. Chitosan-Based Nanomaterials

Chitosan can be obtained by N-deacetylation of chitin, a biopolymer widely used in biomedical fields [73,74]. Due to its low toxicity and good adaptability to cells and tissues, it has been approved in the food and medicine fields. Moreover, the properties of chitosan, which are easy to modify, may lead to its excellent drug delivery ability [75]. Efforts have been made to chemically change active chitosan (e.g., amino groups) for better use in immunotherapy. Currently, lots of chitosan derivatives have been prepared, such as trimethyl chitosan (TMC), carboxymethyl chitosan (CMC), thiolated chitosan (TC), and glycosylated chitosan (GC) (Table 2).

Compared with other carriers, chitosan has the advantage of immunomodulatory ability [76,77]. Chitosan as a drug carrier is also a promising immune adjuvant, and in one study, the application of chitosan significantly increased the response level of humoral and Th1/Th2 cellular immunity [78]. The application of TMC as an immune adjuvant in vaccines was reviewed by Malik et al. [79]. Wen, Z.S. et al. found chitosan could significantly promote the production of tumor suppressor cytokines in mouse spleen cells and enhance tumor killing by NK cells [80].

As early as 2009, Seo, S.H. et al. proposed that the co-delivery of cyclophosphamide (CTX) and GM-CSF into cervical tumors by intratumoral injection of chitosan hydrogel could activate CD8+ T cells in the tumor microenvironment [31]. The researchers also designed a TMC nanoparticle loaded with DOX and rhIL-2 [81]. DOX and TMC are tightly bound by chemical bonds and modified by FA to target tumors effectively. The nano-complex with a positive charge was synthesized by adsorbing recombinant IL-2 on the surface by electrostatic interaction, and its particle size was about 200 nm. The combined therapy has a pronounced antitumor effect and low toxicity in vivo.

Chen, Y. et al. prepared nanoparticles with chitosan as a carrier and encapsulated them in recombinant pcDNA3.1-dsNKG2D-IL-15 [82]. Nanoparticles carrying the dsNKG2D-IL-15 gene can be effectively endocytosed by tumor cells and stimulate the antitumor effects of tumor-killing cells. After treatment, IL-15 levels in tumor tissue were significantly elevated. Similarly, Tan, L. et al. prepared PcDNA3.1-dsNKG2D-IL-21 nanoparticles with chitosan as a carrier [83]. The serum IL-21 level of mice treated with NPs was increased. DsNKG2D-IL-21 gene nanoparticles accumulated in colorectal cancer and lymphoma tissue 4–24 h after intravenous injection. Tumor growth was inhibited by activating NK cells and T cells in vivo, and the survival time of tumor-bearing mice was prolonged.

The above studies show that the nanoparticles prepared by chitosan and its derivatives can effectively transfer cytokines to the target cells, reduce the tumor volume, inhibit tumor activity, and play a role in treating tumors.

### 2.5. Polyethyleneimine-Based Nanomaterials

Polyethyleneimine (PEI) is a hydrophilic cationic polymer with a molecular weight between 1~1000 kDa. The amine functional group in the PEI structure has a pH response and can be used for drug-controlled release [84]. In addition, PEI can be used as a cationic polymer to combine with various negatively charged biomolecules [85]. The advantage of PEI as a drug delivery system is that the positive charge in PEI can easily bind to cell membrane adsorption, thus enhancing immunogenicity and promoting phagocytosis [86]. Like the above polymers, PEI-based nanoparticles have become one of the most popular carriers because of their non-toxicity, non-immunogenicity, and ability to transmit a variety of immunomodulators.

Yim, H. et al. prepared a self-assembled polymeric micellar immunomodulator (SPI) based on a cationic amphiphilic polymer by conjugating all-trans retinoic acid (ATRA) with PEI. It is characteristic of inducing necrosis by recruiting inflammatory cytokines in the return cystic cancer model. Hyaluronic acid (HA) was masked by a cationic charge to form PEI polymer micelles to overcome the necrotic effect. After endocytosis by cells, the positively charged PEI is exposed and stimulates the production of cytokines such as TNF-α, further inhibiting tumor progression [33].

Jiang, J. et al. chose a polymer, β-CD-PEI, with a simple structure and low toxicity but that was functional for combined therapy [34]. CP polymers in the co-administration system promoted cellular uptake of SB-505124, sustained release, and increased adenovirus (Ad) transduction efficiency. CP-SB/Ad-mIL-12 was effective in inhibiting tumor invasion in vitro experiments. In a mouse melanoma model, tumor development was hampered, and survival was prolonged in mice. Elisa and flow cytometry showed that the number of IL-12, T cells, and natural killer cells were significantly elevated after treatment.

### 2.6. Liposomes-Based Nanomaterials

The liposome is a vesicle system with a bilayer phospholipid membrane structure and is a potential drug carrier. Liposomes have been widely used in medical fields because of their protection of drugs, reduction of drug toxicity, histocompatibility, and modifiability. In terms of immunomodulator administration, it has been recently reported that liposome formulations have been updated to improve the effectiveness of drug delivery. For example, polyethylene glycol liposomes with enhanced targeting can effectively deliver loaded drugs to tumor tissue and reduce nanoparticle clearance by macrophages [87]. Currently, efforts are devoted to targeting liposomes, increasing drug loading, and enhancing the uptake of drugs by tumor cells.

Fernandez, M.F. et al. found that the combination of LIGHT (TNFSF14) and IL-2 increased CD8 central memory T cells in vivo [88]. This finding was confirmed in a mouse colorectal cancer tumor model, while the number of tumor-infiltrating lymphocytes (TIL) was not elevated. Liposomes can also enter tumor cells through mRNA encapsulating some cytokines and then regulating TME. Liu, J.Q. et al. encapsulated mRNA encoding IL-12 and IL-27 in liposome nanoparticles [89]. Compared with free drugs, B16F10 melanoma mice injected with IL-12 and IL-27 mRNA liposomes increased the levels of IFN- γ and TNF- α in the tumor and activated NK cells and CD8+T cells. Targeting tumor cells with mRNA-loaded liposomes is a new strategy in cytokine therapy.

## 3. Inorganic Nanomaterials

With the rapid development of organic nanomaterials, researchers have found that several inorganic nanomaterials can carry drugs to transfer or induce cytokines. Furthermore, inorganic nanomaterials’ physical and chemical properties can synergize in cancer immunotherapy. For example, the magnetic properties of Fe_2_O_3_ nanoparticles can be used to enhance immunotherapy. This paper mainly introduces silica nanoparticles, magnetic nanoparticles, and gold nanoparticles. (Table 1)

### 3.1. Silica Nanoparticles

In the past decade or so, mesoporous silica nanoparticles have been widely studied. Mesoporous silica nanoparticles (MSNPs), with the advantages of a larger contact area, a higher drug loading rate, and better modifiability than other nanoparticles. These advantages have led to its importance in the biomedical field. Some drug-loading systems can enhance biocompatibility when combined with silica [90,91,92]. Mesoporous silica nanoparticles can easily adjust the pore size, thus changing the mode of drug delivery. Modified MSNPs are a safe and efficient nanomaterial for targeted tumor therapy [93,94,95].

Liu et al. reported that silica nanoparticles could have a role in promoting humoral immunity [96]. Choi, E.W. et al. investigated the effect of silica NPs loaded with GM-CSF mRNA on dog leukocytes [97]. Kong, M. et al. embedded ATRA, DOX, and IL-2 in hollow MSNPs for immunotherapy of the B16F10 melanoma model [37]. The nanoparticle-mediated combination treatment plays a regulatory role in the tumor microenvironment by activating TILs, promoting the secretion of cytokines, and down-regulating MDSCs. Wan, Y.F. et al. prepared tumor-targeted, microenvironment-responsive mesoporous silica nanoparticles used to wrap IL-12 [38]. Studies have shown that the nanoparticles can effectively target tumor tissue, be swallowed by macrophages, release IL-12 locally, and repolarize TAM to an M1 phenotype that can kill the tumor with fewer side effects.

### 3.2. Magnetic Nanoparticles

Magnetic nanoparticles (MNPs) are mainly based on Fe_2_O_3_ and Fe_3_O_4_. Because of the biocompatibility and modifiability they possess, these nanoparticles are well-suited as drug delivery platforms for drug delivery. In addition, the magnetism of MNPs provides an essential ability to transmit heat [98]. The magnetic properties of MNPs make them uniquely suited to receive magnetic field stimulation in vitro, a property that can be used for immune enhancement. 

Mejías, R. et al. used dimercaptosuccinic acid (DMSA) for surface modification of Fe_2_O_3_ NPs. They studied the inhibitory effect on tumors after adsorption of IFN-γ in a mouse pancreatic ductal adenocarcinoma model [39]. The nanoparticles enabled efficient drug delivery and tumor enrichment with a substantial increase in T cells and macrophages for effective tumor suppression. Hu, B. et al. prepared anti-cancer magnetic polymer microspheres T9-TNF-PC-M containing human transferrin receptor monoclonal antibody (T9), TNF, and Fe_3_O_4_ ultrafine magnetic powder (M) by solvent evaporation method [40]. The T9-TNF-PC-M has a stable release rate of tumor necrosis factor, a solid magnetic response, and high drug loading in phosphate-buffered saline solution. The cytotoxicity test in vitro showed that T9-TNF-PC-M and their conjugates strongly inhibited human hepatocellular carcinoma cells. The in vivo targeted therapy showed that the antitumor activity of microsphere T9-TNF-PC-M and T9-TNF against Bel-7204 was significantly higher than that of free tumor necrosis factor. Ye, H. et al. synthesized magnetic liposomes containing recombinant human interferon-α2β (MIL) by combining the magnetic nanomaterial Fe_3_O_4_ with liposomes and evaluated the biosafety and therapeutic effect of this combination on cells and hepatocellular carcinoma in mice [41]. The results show that MIL can neither dissolve red blood cells nor affect the platelet aggregation rate in blood. The nanoparticles effectively prolonged the drug action time by applying a magnetic field externally. MIL significantly inhibits the development of hepatocellular carcinoma cells. The targeting experiment of MIL showed that MIL could considerably reduce the tumor volume of nude mice, which was 38% of that of the control group.

### 3.3. Gold Nanoparticles

Gold nanoparticles (GNPs) are widely explored because of their excellent prospects in nanotechnology, especially in biological nanotechnology for detection, imaging, and therapy [99]. Colloidal gold was commonly used in treating various diseases, primarily because of its optical properties and magnetism. GNPs have low toxicity and good biocompatibility, which benefits their interaction with other biomolecules [100]. GNPs are increasingly used in clinical research because they are easy to synthesize and process [101]. Gold is usually designed as nanoparticles, nanocages, nanoshells, nanostars, nanorods, and so on [102]. 

A team from Milan, Italy, has developed a drug delivery platform that enhances tumor targeting by modifying gold nanoparticles to deliver cytokines to tumor cells [42,103,104]. One of the gold nanoparticles labeled with a novel tumor-homing peptide containing the CD13 ligand ASN-Gly-Arg (NGR) expressed in tumor neovascularization can be used as a carrier for the delivery of cytokines to tumors [42]. In mice with fibrosarcoma, NGR-labeled nanoparticles can deliver very low but pharmacologically active levels of TNF to cancer. This experiment shows that NGR-labeled gold nanoparticles can be treated as a new platform, enhancing drug delivery targeting.

Mohseni, N. et al. developed a gold nanorod coupled with interferon-γ and methionine combined with near-infrared laser hyperthermia, which can be used to treat tumors [43]. Different concentrations of GNPs were added to cultured breast cancer cells and irradiated using NIR light. In the process of NIR light irradiation, the number of tumor cell deaths in the presence of GNPs was significantly higher.

### 3.4. Calcium Carbonate/Calcium Phosphate Nanoparticles

CaCO_3_ and Ca_3_(PO_4_)_2_ nanoparticles are suitable drug carriers with good biosafety and degradability and have already been used in tissue engineering and drug delivery [105]. Because of their responsiveness to the acidic tumor microenvironment, CaCO_3_ and Ca_3_(PO_4_)_2_ nanoparticles are well-suited drug delivery systems for tumor immunotherapy [106].

Liu et al. prepared calcium carbonate nanoparticles loaded with shiitake mushroom polysaccharides, which could be treated as immune adjuvants to strengthen cellular and humoral immune responses. In a tumor model, the NPs induced the secretion of IL-2 and IL-4 [44]. Mao et al. prepared M-CSF-loaded CaCO_3_ nano micelles that were pH-responsive to the tumor microenvironment and could effectively target C57BL/6 mouse melanoma tissue and release M-CSF to enhance the antitumor effects of macrophages and T cells [45]. Chen et al. used CaCO_3_ nanogels with anti-CD47 antibodies to prevent local murine melanoma tumor recurrence and to improve macrophage phagocytosis and antigen presentation by postoperative in situ spraying [107].

## 4. Novel Nano Delivery Systems

With the development of nanomaterials and cytokine therapies, a series of novel nano-delivery systems have emerged in recent years. Hybridized nanoparticles have come into the limelight due to their ability to combine various types of nanoparticles. By combining two or more nanomaterials, the nanoparticles can synergize with cytokines to enhance tumor immunotherapy. Zhang et al. prepared lipid-polymer nanoparticles synthesized by PCL-PEG-PCL and DOTAP (IMNPs). The nanoparticles loaded with TLR 7/8 agonist and TLR4 agonist monophosphoryl lipid A (MPLA) could effectively target DC cells to suppress tumors and prolong survival in mice [108]. Gao et al. developed a Gd-Au DENP-PS nanoplatform for encapsulating PD-1 siRNA. The nanoparticles are dendritic molecules that activate T cells and bind to IDO to enhance tumor immunotherapy [109]. A manganese-based hybrid nanoparticle was also reported. Using amorphous porous manganese phosphate (APMP) loaded with DOX and phospholipids (PL), Hou et al. The drug enhanced cellular immune response and tumor-killer cell recruitment while increasing the secretion of cytokines, acting as an antitumor agent [110].

**Figure 2:**
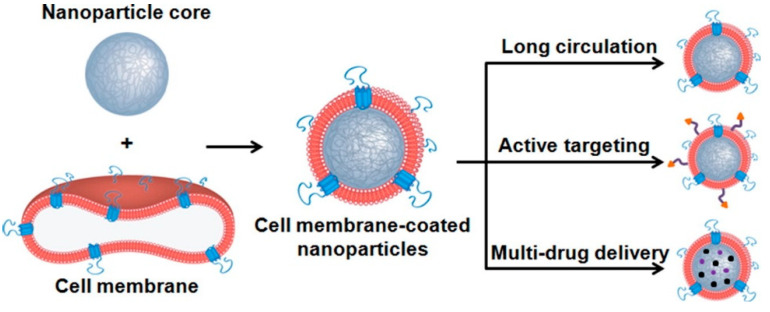
Diagram of membrane camouflaged nanoparticles. Adapted from Brian, T.L. et al., 2015 [118].

Membrane camouflage nanoparticles (MCNPs), usually derived from erythrocytes, cancer cells, neutrophils, and platelets, are potential drug delivery platforms because of their immune advantages. These materials can often avoid drug clearance in vivo and target tumor cells more effectively, and they have a high affinity for cells of the exact origin [111,112]. Erythrocyte membrane is the most common source of MCNPs due to its ease of obtaining, excellent biocompatibility, and strong protection for loaded drugs [113]. Cancer cell membrane nanoparticles have the following distinctive characteristics: they cannot be easily removed; adhesion molecules on the membranes can promote the homologous targeting of cancer cells; and CD47 on the membrane of cancer cells prevents phagocytes from engulfing nanoparticles [114,115]. Platelet membrane is rich in sources, has potential camouflage to evade immune surveillance, and avoids the circulatory release of drugs, which has become an ideal material for biomimetic carriers. Therefore, developing nanoparticles camouflaged by platelet membranes is a promising research direction [116]. Neutrophil membranes are also unique because they have a variety of cytokine receptors on their surface [117]. In summary, the excellent camouflage and good biocompatibility provided by cell membranes will have significant advantages in competing with other immunomodulator delivery technologies (Figure 2).

Similarly, because of its outstanding biocompatibility, the cell-based nano-drug delivery system is also a new way of drug delivery, such as nanosystems based on red blood cells (RBC) [119], nanosystems based on immune cells [117], nanosystems based on stem cells [117], and nanosystems based on platelets [120]. This kind of system will deliver cytokines to tumor cells more efficiently. Therefore, more attention should be paid to applying cytokine drugs to cell-based nanosystems.

NPs based on phototherapy have the advantages of a solid curative effect, low invasion, and few adverse reactions in tumor therapy. Photothermal therapy (PTT) and photodynamic therapy (PDT) are the two primary treatment modalities of phototherapy. PTT and PDT based on nanoparticles can kill tumors directly and induce continuous antitumor immune effects. PTT uses heat to kill tumors, while PDT kills tumors by producing large amounts of ROS in tumor cells. In particular, the death of many tumor cells after PTT and PDT results in a more intense immune response, including reprogramming and activation of the immune microenvironment, modulation of cytokines, and mediation of a more intense T-cell immune response [121,122,123,124,125]. If phototherapy can be combined with existing cytokine immunotherapy, it will become a more efficient method for tumor treatment.

## 5. Discussion and Conclusions

The various nano-drug delivery techniques discussed above can effectively use cytokines in cancer immunotherapy and solve the problem of poor curative effects due to the limitations of cytokines. In the last few years, with the advancement of nanotechnology, many emerging drug delivery systems have emerged, which provide a new idea for the delivery of cytokines into tumor cells. However, nano-drug delivery systems also have unpredictable risks, such as “hand and foot syndrome” in cancer patients treated with doses of statin [126]. Although targeted delivery of immunomodulatory drugs through nanomaterials has dramatically improved the delivery efficiency, with the deepening of treatment, these nanoparticles inevitably accumulate in vivo and produce an unpredictable adverse reaction. In addition, for most or all delivery systems, the cost of materials and the feasibility of mass production have to be considered, as demonstrated by Doxil’s repeated severe supply shortages [127,128,129]. 

In the combined treatment of traditional chemotherapeutic drugs, some differences make it difficult for the drugs with synergistic effects to be simultaneously loaded on a single drug delivery platform. So, solving the typical delivery of hydrophilic and hydrophobic drugs is still a complex problem. For example, the co-loading of hydrophilic IL-12 and hydrophobic PTX is a problem that needs to be solved [130]. To explore the method of co-delivery between IL-12 and PTX, Sun, C.Y. et al. designed a pH-sensitive material, mPEG-Dlinkm-PDLLA. The mPEG-Dlinkm-PDLLA copolymer-loaded drug cannot be released into the blood. The mPEG-Dlinkm-PDLLA copolymer-loaded drug is not released into the blood. In the tumor microenvironment, pH-sensitive chemical bonds are broken, and the drug is released into the tumor microenvironment, acting as a modulator of tumor immunity [130]. The use of immune adjuvants to enhance the effect of immunotherapy is also an option that can be considered. Both protamine and bovine serum albumin can enhance immunity and combine with nanomaterials to enhance the efficacy of immunotherapy [131].

In the past decade, immunotherapy has occupied a higher and higher proportion in the treatment of cancer [132]. Meanwhile, combining immunotherapy with other treatments is also being explored step by step. In future research, the combination of the nanotechnologies mentioned above and other traditional therapies should be considered in treating tumors. Yoshida, Y. et al. studied the feasibility of immunotherapy combined with chemotherapy in treating stage IV colon cancer. This therapy uses αβ T cells cultured with CD3 and IL-2, whose toxicity in vivo often affects the effect of cancer treatment [133].

Although cytokines have made some achievements in cancer therapy, their efficacy is still minimal. One possible reason is that cytokines have a short half-life in the blood. High doses are usually needed to achieve lasting therapeutic effects. Despite the achievements of cytokines as therapeutic agents, the therapeutic effect is still not satisfactory, probably because cytokines are primarily proteins that may be affected by various factors in the body [13]. Therefore, the current strategy is mainly to use high-dose cytokine therapy. Patients treated with cytokines often have adverse reactions, including fatigue, chills, fever, chest pain, and musculoskeletal pain [134]. Gastrointestinal disorders, cardiac abnormalities, immune system disorders, and blood and lymphatic system disorders are more severe side effects. Using the appropriate nano-delivery system to deliver cytokines can achieve a better therapeutic effect and has a broad prospect in combination with other therapies [135]. Therefore, the problems we need to solve in the future should include the rationality of chemical modification, drug loading, stability, cell internalization, and different physical and chemical properties of drugs. The design of nano drug delivery systems should consider the material’s protection, and the clearance in the liver and kidneys should be reduced, as should the choice of drug delivery method and the enhancement of targeting to tumor tissues [136]. At the same time, we should fully use the characteristics of nanomaterials, such as magnetic nanoparticles and the immune enhancement properties of chitosan nanoparticles, to enhance the effect of immunotherapy. In addition, attention should be paid to the toxicity and side effects of nano-delivery systems, and the cost should be reduced as much as possible to achieve mass production. 

## Figures and Tables

**Table 1:** Classification of nanomaterials and cytokines involved in the regulation.

	Nanomaterials	Cytokines	References
Organic	PLGA-based nanomaterials	TNF-α, IL-6, IFN-α,GM-CSF	[22,23,24]
	Poly-γ-glutamic acid-based nanomaterials	IL-10, IL-12, IL-6,TNF-α, IFN-γ	[25,26]
	β-Cyclodextrin-based nanomaterials	VEGF, IL-10, IL-12	[27,28,29,30]
	Chitosan-based nanomaterials	IL-2, IL-12, IL-15, IL-21	[31,32]
	Polyethyleneimine-based nanomaterials	IL-6, TNF-α, IL-12, IFN-γ	[33,34]
	Liposomes-based nanomaterials	IL-2, TGF-β	[35,36]
Inorganic	Silica nanoparticles	IL-2, IFN-γ, IL-12	[37,38]
	Magnetic nanoparticles	IFN-γ, TNF-α, IFN-α	[39,40,41]
	Gold nanoparticles	TNF-α, IFN-γ	[39,42,43]
	Calcium carbonate/Calcium phosphate nanoparticles	IL-2, IL-4, M-CSF	[42,44,45]

**Table 2:** The structures of chitosan and its properties.

Type	Chemical Structure	Properties
Chitosan	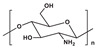	Nonimmunogenic; Biocompatible; Biodegradable; Mucoadhesion;
Trimethyl chitosan (TMC)	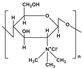	Positively charged; Penetration-enhancement; Mucoadhesion;
Carboxymethyl chitosan (CMC)	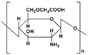	pH-dependent water solubility; Adhesion and absorption enhancement; Antibacterial and antioxidant activities;
Thiolated chitosan (TC)	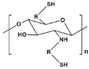	Easily endocytosed by cells; Hydrophilic; In situ relatability; Mechanical stability;
Glycated chitosan (GC)	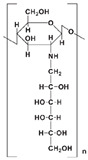	Immune-enhancing; Hydrophilic; Noncytotoxic;

## Data Availability

Not applicable.
